# Monitoring Spatial Variability and Temporal Dynamics of *Phragmites* Using Unmanned Aerial Vehicles

**DOI:** 10.3389/fpls.2018.00728

**Published:** 2018-06-04

**Authors:** Viktor R. Tóth

**Affiliations:** Balaton Limnological Institute, MTA Centre for Ecological Research, Tihany, Hungary

**Keywords:** UAV, NDVI, phenology, macrophyte, die-back, mowing

## Abstract

Littoral zones of freshwater lakes are exposed to environmental impacts from both terrestrial and aquatic sides, while substantial anthropogenic pressure also affects the high spatial, and temporal variability of the ecotone. In this study, the possibility of monitoring seasonal and spatial changes in reed (*Phragmites australis*) stands using an unmanned aerial vehicle (UAV) based remote sensing technique was examined. Stands in eutrophic and mesotrophic parts of Lake Balaton including not deteriorating (stable) and deteriorating (die-back) patches, were tracked throughout the growing season using a UAV equipped with a Normalized Difference Vegetation Index (NDVI) camera. Photophysiological parameters of *P. australis* were also measured with amplitude modulated fluorescence. Parameters characterizing the dynamics of seasonal changes in NDVI data were used for phenological comparison of eutrophic and mesotrophic, stable and die-back, terrestrial and aquatic, mowed and not-mowed patches of reed. It was shown that stable *Phragmites* plants from the eutrophic part of the lake reached specific phenological stages up to 3.5 days earlier than plants from the mesotrophic part of the lake. The phenological changes correlated with trophic (total and nitrate-nitrite nitrogen) and physical (organic C and clay content) properties of the sediment, while only minor relationships with air and water temperature were found. Phenological differences between the stable and die-back stands were even more pronounced, with ~34% higher rates of NDVI increase in stable than die-back patches, while the period of NDVI increase was 16 days longer. Aquatic and terrestrial parts of reed stands showed no phenological differences, although intermediate areas (shallow water parts of stands) were found to be less vigorous. Winter mowing of dried *Phragmites* sped up sprouting and growth of reed in the spring. This study showed that remote sensing-derived photophysiological and phenological variability within and between reed stands may provide valuable early indicators of environmental stress. The flexibility of the method makes it usable for mapping fine-scale temporal variability and spatial zonation within a stand, revealing ecophysiological hotspots that might require particular attention, and obtaining information vital for conservation and management of plants in the littoral zones.

## Introduction

Lakes throughout the world are exposed to many abiotic, biotic, and anthropogenic stressors originating from both the aquatic, as well as the adjacent terrestrial environment. Impacts on lakes from the terrestrial ecosystem first affect the shoreline areas inhabited by macroscopic vascular plants, the littoral zone (Phillips et al., 2016; Vermaat et al., 2016), which acts as a buffer. This protective ecosystem and socioeconomic service provided by the littoral zone also endangers it, making the assessment and protection of these areas vital. Characteristics of freshwater lakes' littoral zones include their wide spatial extent, high primary productivity and biomass, and high biodiversity. The highest biodiversity of the entire lake system is sometimes found here, especially in shallow freshwater lakes (Strayer and Findlay, 2010), as a result of the environmental heterogeneity of this ecotone (Schmieder, 2004; Meerhoff et al., 2007). Littoral zones maintain this biodiversity and variability despite the constant pressures from both the aquatic and terrestrial ecosystems (Radomski and Goeman, 2001; Strayer and Findlay, 2010). Macrophytes are the defining component of the littoral zone, are involved in multiple ecosystem services, are vital biotic substrates for large numbers of organisms, play a pivotal role in element cycling, and provide habitats with diverse food, cover, and structure for the aquatic biota (Wetzel, 1975; Wigand et al., 1997).

Monitoring the macrovegetation of the littoral zone is important, since either the loss or proliferation of aquatic plants may trigger unwanted ecosystem shifts, resulting in biodiversity loss, habitat degradation, and further ecosystem changes (Scheffer et al., 1993; Jeppesen et al., 1998). Scientists study the littoral zone from various angles, carrying out *in situ* measurements, laboratory analyses, and also using Earth Observation for assessments of both water quality and vegetation health and coverage (Brezonik et al., 2015; Palmer et al., 2015; Villa et al., 2017). The latest generation of airborne, spaceborne, and Light Detection and Ranging (LIDAR) imaging sensors can be effectively exploited to map relevant aspects of the water surface and important vegetation features based on their respective spectral responses (Hunter et al., 2010; Zlinszky et al., 2012; Villa et al., 2013; Stratoulias et al., 2015). Although these methods are useful, important spatial, and temporal patterns may be overlooked due to their coarse spatiotemporal resolution. Unmanned Aerial Vehicle (UAV) platforms combine several advantages of airborne and spaceborne remote sensing with the flexibility of autonomous operation such that the user can rapidly respond to meteorological and other conditions. Moreover, as a versatile platform, UAVs can be equipped with a diversity of sensors corresponding to the particular objectives of the mission.

To estimate the changes within an ecosystem, to evaluate the state of its biodiversity and thus help the ecosystem management requires not only a single time, but rather a continuous assessment (change detection, classification, projection) of the study area. Remote sensing (especially the satellite) proved to be a useful and versatile tool to measure and calculate ecosystem dynamics and vegetation indices mostly related to plant biometrics were successfully used to estimate vegetation parameters over time (Reed et al., 1994; Fisher and Mustard, 2007; Luo et al., 2016).

On an intra-annual timescale plants show recurring, very specific phenomena at well-defined times. These sequence of events (i.e., phenology) could be used to describe plant species. Traditionally plant phenology used discrete descriptors as timing of emergence, timing of flowering, start, and end of senescence. More recently, the use of remote sensing and vegetation indices like Normalized Difference Vegetation Index (NDVI) have helped the phenological studies (Jönsson and Eklundh, 2004; Pettorelli et al., 2005). NDVI approximates the photosynthetically active leaf area, since plant leaves absorb red (RED), and reflect near infrared (NIR) wavelengths, thus a RED to NIR reflectance ratio ($NDVI=\frac{\left(NIR-RED\right)}{\left(NIR+RED\right)}$) amounts for leaf biomass (Asrar et al., 1984; Sellers et al., 1992). Thus, time series of NDVI data could show vegetation dynamics along season, i.e., a unimodal temporal distribution of plant biomass that in the temperate zone is driven by temperature and is closely related to phenology. The unimodal trajectory of vegetation dynamics consists of accumulation and later decomposition of plant biomass, but for simplicity of calculations, in this study only the ascending (accumulation) part of the distribution was used. From it, the timing of maximal NDVI intensity growth, the NDVI maximum of the season and the initial rate of NDVI increase were calculated and used as phenological descriptors.

First objective of this study was to explore the use of UAVs for high resolution remote sensing of emergent aquatic plants. The sites along the difficult-to-access areas of Lake Balaton's littoral zone could demonstrate the viability of this technique to support wetland and macrophyte research, as well as the management of these stands in particular, and vegetation studies more generally. Second objective was to assess the feasibility of using UAVs to investigate plant phenology, based on the example of *Phragmites* in Lake Balaton (i.e., whether it is possible to perform repeated measurements on the same spot throughout the vegetation period). The third objective was to assess whether a UAV platform can be used for high definition survey and thematic (photophysiological) mapping of wetlands. To this end, an empirical model to map macrophyte photophysiological traits using NDVI was elaborated and applied.

## Materials and methods

### Study sites

**Figure 1:**
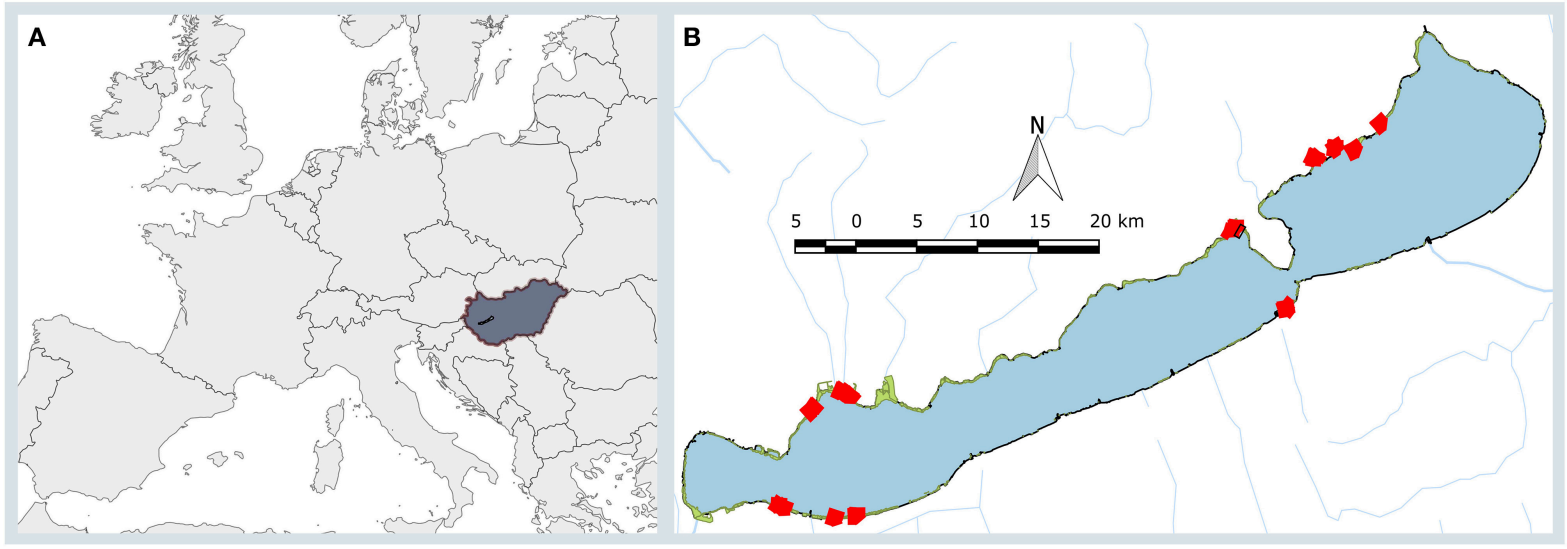
**(A)**. The map of the Europe (gray) showing Hungary (dark gray, brown outline) and Lake Balaton (black) within it. **(B)**. Lake Balaton with its smaller tributaries (blue lines) and its reed stands (green areas) and the sampling points (red squares). Black square shows the position of the area depicted on Figure 2.

Lake Balaton (Figure 1), the largest freshwater lake of Central Europe, is 78 km long, 6 km wide (596 km^2^), and relatively shallow (average water depth is 3.5 m). Nearly half of its 240 km shoreline is semi-natural, and mostly covered with wetlands as large as 13.6 km^2^ that are sometimes inaccessible. The dominant species of these wetland areas is *Phragmites australis (Cav.) Trin. ex Steud*, which is commonly found in extended, continuous populations.

*Phragmites* patches in the western (eutrophic) and eastern (mesotrophic) areas of Lake Balaton were chosen (Figure 1, Supplementary Table 1). The shortest distance between the studied western and eastern patches was 33 km, while the largest was 59 km. The majority of the stands (21) were in stable (not deteriorating) condition, although, four sites characterized by die-back of reed, were also part of the study. This differentiation between stable and die-back stands was made on morphological and phytocoenological analysis made in previous studies (Tóth and Szabó, 2012; Tóth, 2016). Two further study areas were included, because these stands were mowed during the winter, extending the variability of the selected sites (Figure 1, Supplementary Table 1), while 23 patches were untouched (not mowed) for at least 5 years (Supplementary Table 1), thus contained dry reed stems from previous seasons.

**Figure 2:**
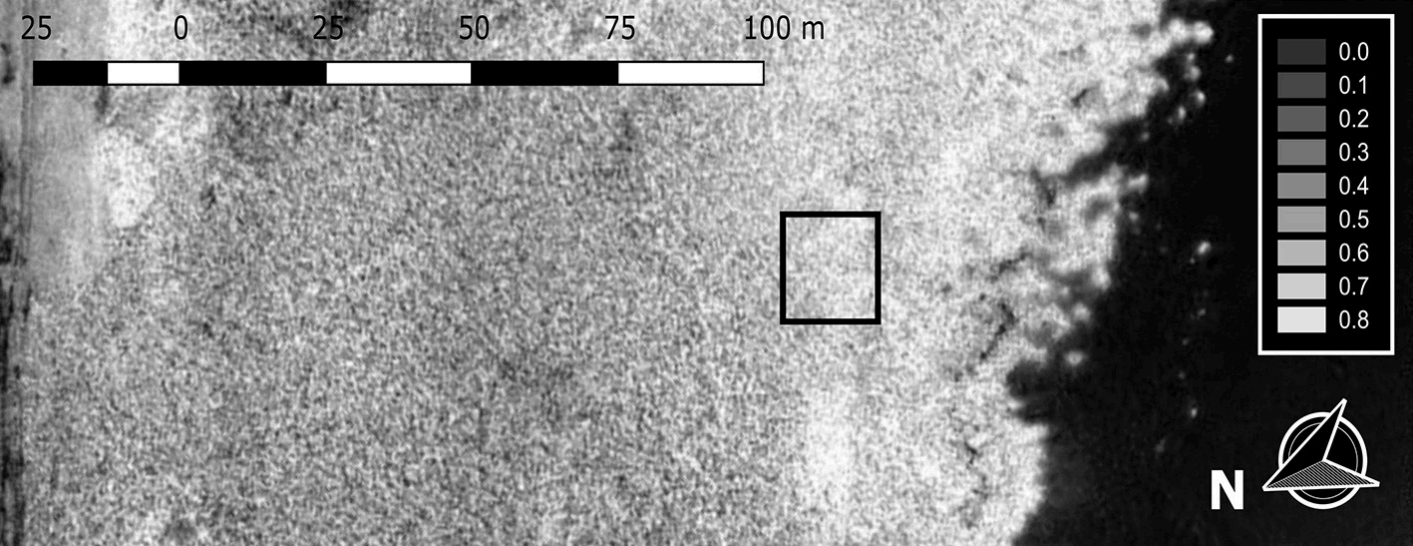
Orthomosaic image of the normalized difference vegetation index (NDVI) values of a 140 m long reed stand in the eastern part of Lake Balaton. NDVI was captured from altitude of 100 m with original ground resolution 40 mm per pixel. The black area on the right depicts the water of Lake Balaton. The black quadrate indicates the study area.

The study areas were ~100 m^2^ patches of monodominant *Phragmites*. The stable (non-degrading) patches were homogeneous, with ~100% cover (Figure 2), while the patches at the die-back (degrading) sites were inhomogeneous, with average cover of ~80% (Supplementary Table 1). All of the studied patches (both stable and die-back) were situated 20–30 m from the lakeward side of the reed stands, thus on the northern shore the plants were in 100–120 cm, while on the southern shore in 60–80 cm deep water during the whole vegetation period.

### NDVI analyses

At least once a month during the vegetation period (April-October 2015), NDVI was determined at all studied *Phragmites* patches. NDVI was measured using an NDVI camera (ADC Lite, Tetracam, US) mounted on a UAV platform consisting of an MK Okto XL 6S12 (MikroKopter, Germany) with MC-32 HoTT Transmitter PRO. The platform was equipped with an external remote trigger for the camera. The NDVI camera has a 3.2 megapixel CMOS sensor behind a 8.0 mm focal length camera lens. Images were recorded in the red, green, and near infrared (NIR) spectra, with color separation with a Bayer filter and respective spectral maxima at 590, 660 and 800 nm. During favorable weather conditions (low winds, no cloud cover), as many flights as possible were performed within 1 day between the hours of 10h00 and 15h00 local time, corresponding with adequate sun height. To increase the comparability of the data, each site was surveyed nearly at the same time of the day. NDVI data recorded during these flights were retrieved from the camera and assessed with the software PixelWrench2. TO prevent necessity of orthocorrection, during the retrieval of the data only the central part (1/9th) of the NDVI image was used. Please see Supplementary Table 1 for more information on number of measurements and places.

**Figure 3:**
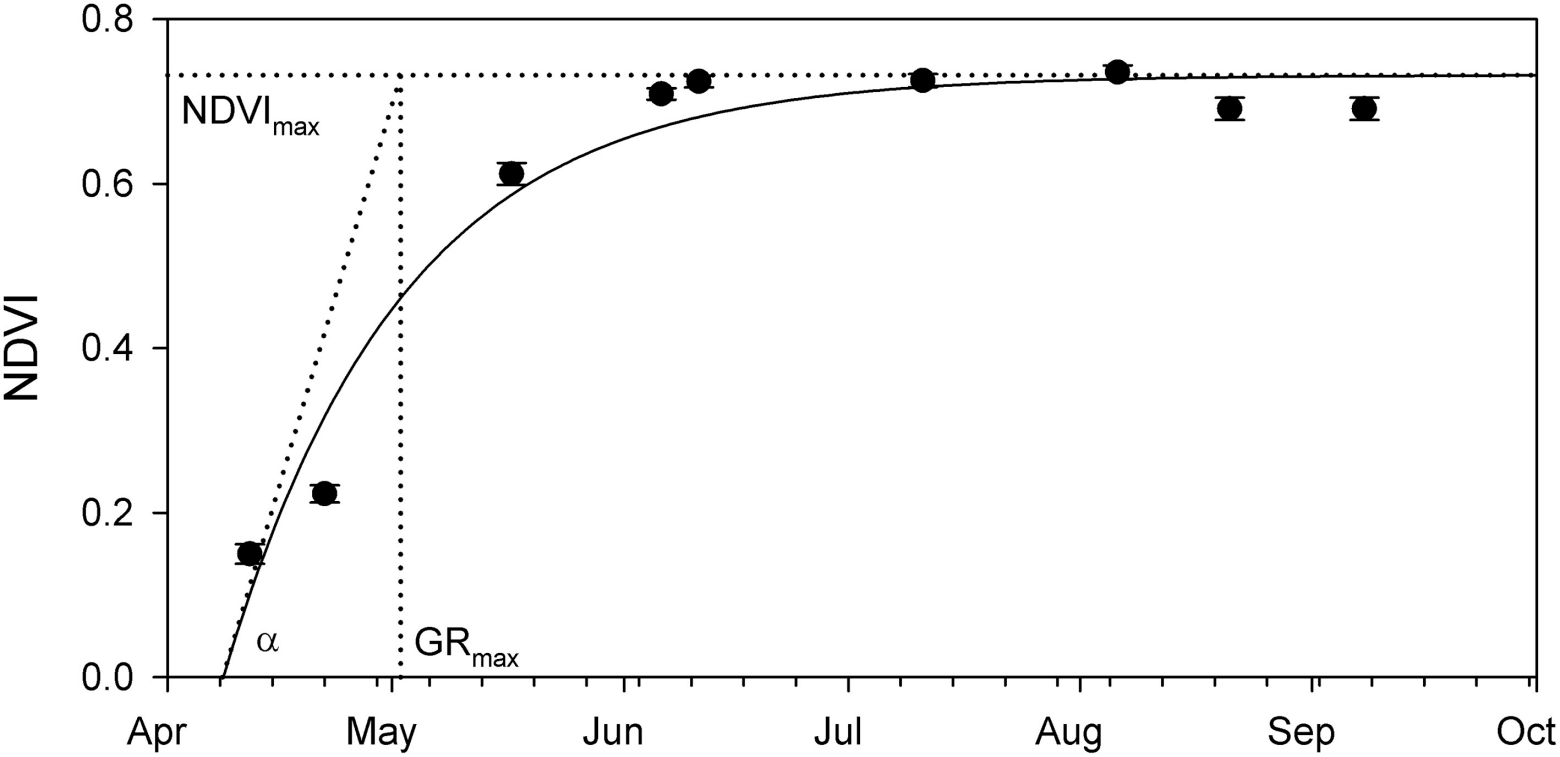
Example of the seasonal change of the NDVI measured at one of the *Phragmites* stands in Lake Balaton. Fitted exponential saturation equation resulted in the NDVI_max_–seasonal maximal NDVI value, GR_max_–day of the maximal NDVI growth, and α–theoretical maximal NDVI growth rate. Symbols show average ± SE (*N* = 1,200 pixels).

Phenology graphs based on NDVI data were produced for each study site separately: an exponential saturating equation was fit to the data (Figure 3), and the retrieved NDVI_max_, GR_max_, and α were used to describe and compare the dynamics of vegetation growth at the studied sites.

The changing GPS accuracy made revisiting the same area with the UAV platform difficult. Accuracy of the UAV's GPS depended on a combination of different physical and environmental factors that vary on daily basis. Therefore, after the nine measurements during a season, due to overlap the original individual ~1,000 m^2^ sampling area was reduced to 2–18 m^2^. The pixels from the given area were averaged, and were used for comparison. Please see supplemental material for camera ground pixel size, number of pixels used in statistics.

On four (two stable and two die-back) stands not point, but transect measurements were performed using the same UAV platform and NDVI camera. The flight was pre-programmed to have at least 30% overlap between each shot. From these individual shots NDVI orthomosaics were created using VisualSFM (http://ccwu.me/vsfm/) and CMVS (http://www.di.ens.fr/cmvs/). Scale invariant feature transform matched all the images pairwise and a 3d image cloud was created. Using CMVS a dense 3d reconstruction was performed that resulted an orthomosaic image of.

NDVI data were validated using an HR2000+ (Ocean Optics, USA) spectroradiometer. Validation was performed along the vegetation season at the terrestrial side of several studied reed stands with nearly 100% cover: following an NDVI camera shot a canopy spectroradiometric measurement was performed and the calculated and measured NDVI indices were compared (see Supplementary Figure 1).

### NDVI seasonality metrics

Seasonal changes in NDVI values of *Phragmites* at each site were fitted with an exponential growth till maximum equation ($y=y_{0}+a\left(1-e^{-bx}\right)$), with *P* < 0.01. For each fit, the following parameters were calculated:

- the maximal seasonal NDVI: *NDVI*_max_ = *a* + *y*_0_
- the time of maximal NDVI intensity increase: $GR_{\max}=\frac{1-\ln\left(\frac{a+y_{0}}{a}\right)}{b}$
- the initial rate of NDVI increase: $\alpha =\frac{NDVI_{\max}}{GR_{\max}}=\frac{\left(a+y_{0}\right)*b}{1-ln\left(\frac{a+y_{0}}{a}\right)}$.

### Morphological analyses

To assess seasonal development of plants, at four sites (2 stable and 2 die-back stands, one each in the western and eastern parts of Lake Balaton) *Phragmites* plants were sampled 1 day following the NDVI image acquisition. At each sampling time in the close proximity of the studied patches at least 10 random plants were cut at their connection to their vertical rhizomes (sometimes under the water level), and several biometric measurements were performed: plant height, measured from the cut surface to the tip of the top leaf; diameter, measured in the middle of the most basal internode of each cut stem, to the nearest 0.1 mm with a vernier caliper; and green leaf counts (number per cut plant).

### Sediment analyses

To estimate abiotic differences between the sites, sediment sampling was performed in the vicinity of seven of the studied patches, four from the eastern part and three from the western part of the lake. Sediment samples were collected in 500 mm long, 60 mm (53 mm inner) diameter plastic tubes. The contents of the tube were mixed and used to determine chemical and physical parameters of the sediment. N-forms — ammonium, nitrate, and urea — were determined following the methods of Mackereth et al. (1978), Elliott and Porter (1971), and Newell et al. (1967), respectively. The total soluble phosphorus (TSP) was determined by the modified method of Gales et al. (1966). Organic C content of the sediment was determined gravimetrically as ignition loss following gradual heating to 550°C (CaCO_3_ content of the samples was taken into consideration). The water capacity of the sediment samples was measured as the upper limit of plasticity of the dried and then re-watered samples with the following typical texture classes:

**Table:** 

Coarse sand	<25 ml 10^2^ g^−1^ sediment
Sand	25–30 ml 10^2^ g^−1^ sediment
Sandy loam	31–37 ml 10^2^ g^−1^ sediment
Loam	38–42 ml 10^2^ g^−1^ sediment
Clay loam	43–60 ml 10^2^ g^−1^ sediment
Clay	51–60 ml 10^2^ g^−1^ sediment
Heavy clay	81–90 ml 10^2^ g^−1^ sediment.

### Fluorescence measurements

Chlorophyll fluorescence was performed at seven of the studied patches (see supplement, four from the eastern part and three from the western part of the lake). Light response curves [i.e., the electron transport rate (ETR) of the photosystem II (PSII) as a function of photosynthetically active radiation (PAR)] of the most apical, fully mature leaf was determined on five *Phragmites* plants at each site using PAM-2500 (Heinz Walz GmbH, Germany) following a dark-adapting period of 20 min. First, the ETR value was determined for a dark-adapted leaf with a pulse of saturated light (630 nm, intensity 3000 μmol m^−2^ s^−1^). Then, the measured leaves were exposed to 11 actinic lights (duration 15 s, 630 nm, intensity between 5 and 787 μmol m^−2^ s^−1^) and the ETR values were measured after each illumination step with a new pulse of saturated (3000 μmol m^−2^ s^−1^) light. The light response data were fitted with an exponential curve (Eilers and Peeters, 1988), and the maximal electron transport rate (ETR_max_), theoretical saturation light intensity (I_k_), and the maximum quantum yield for whole chain electron transport (α) were retrieved from this formula.

### Statistical analyses

The seasonal change of *Phragmites* stem lengths at each sampling site were fitted with a logistic equation ($y=\frac{a}{\left(1+e^{-b\left(x-x_{0}\right)}\right)}$), with *P* < 0.01. For each fit, the following parameters were calculated:

- the date of the peak stem growth (***b*** [day of the year]),
- the intensity of growth (first derivative of the logistic equation [cm d^−1^]).

The seasonal change of green leaf numbers also were fitted with an exponential growth till maximum equation (see above).

Graphing, curve fitting, and statistics were performed in SigmaPlot 12.5 and RExcel v.3.0.17 (Baier and Neuwirth, 2007).

## Results

In general, the temporal changes of the stable *Phragmites* stands' NDVI were similar across all studied plots: positive NDVI values were recorded in the middle of April, while maximal NDVI increase (GR_max_) was measured on the 2 May (day 121.0 ± 0.8), resulting in a seasonal maximum NDVI (NDVI_max_) of 0.7 ± 0.0.

### Western–eastern comparison

**Figure 4:**
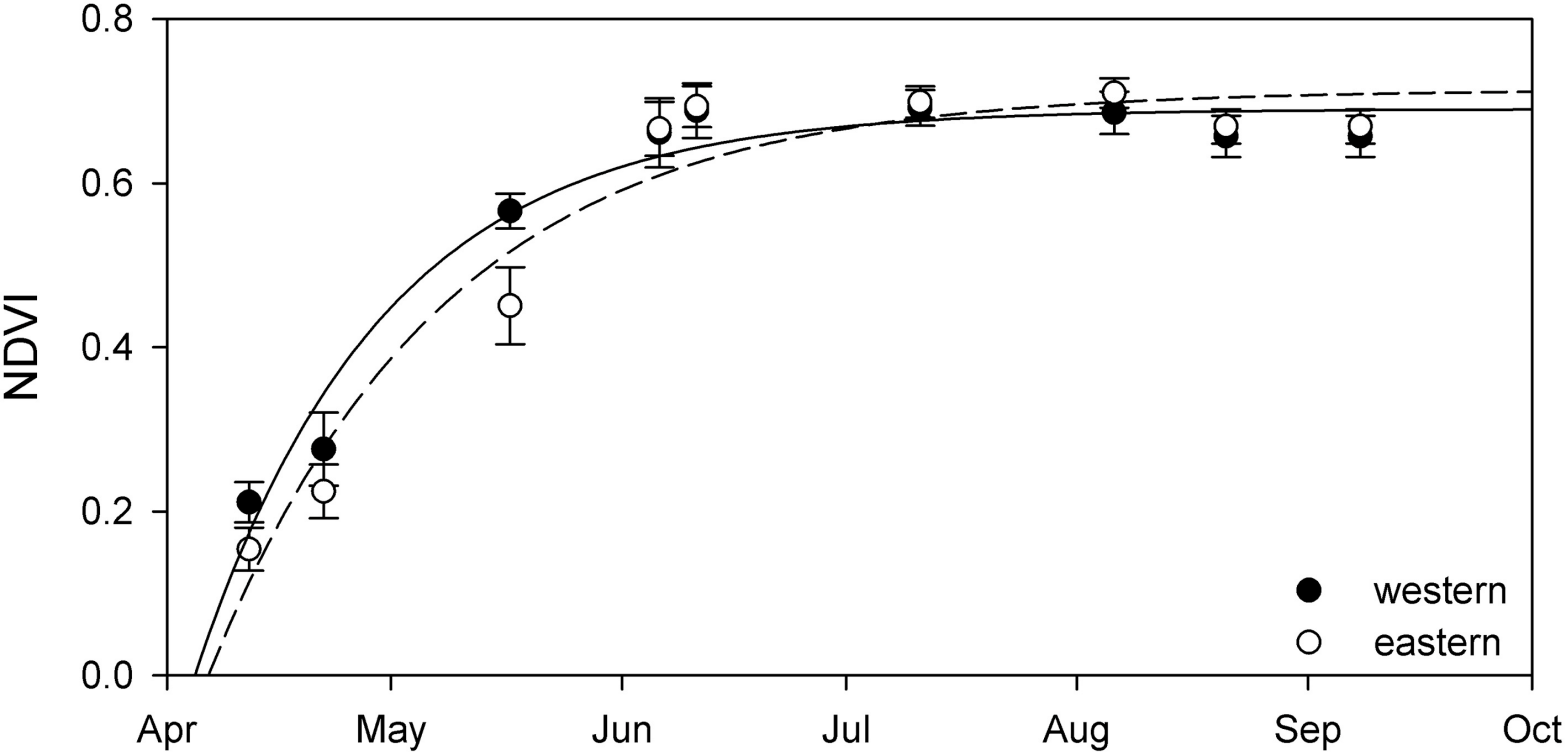
Seasonal change of NDVI of stable *Phragmites australis* plants from the western (black circles) and eastern (open circles) parts of Lake Balaton. Each symbol represents an average (±SE, *n* = 9,100 pixels for western, *n* = 12,100 pixels for eastern). Lines are exponential growth till maximum equation fitted to data.

**Figure 5:**
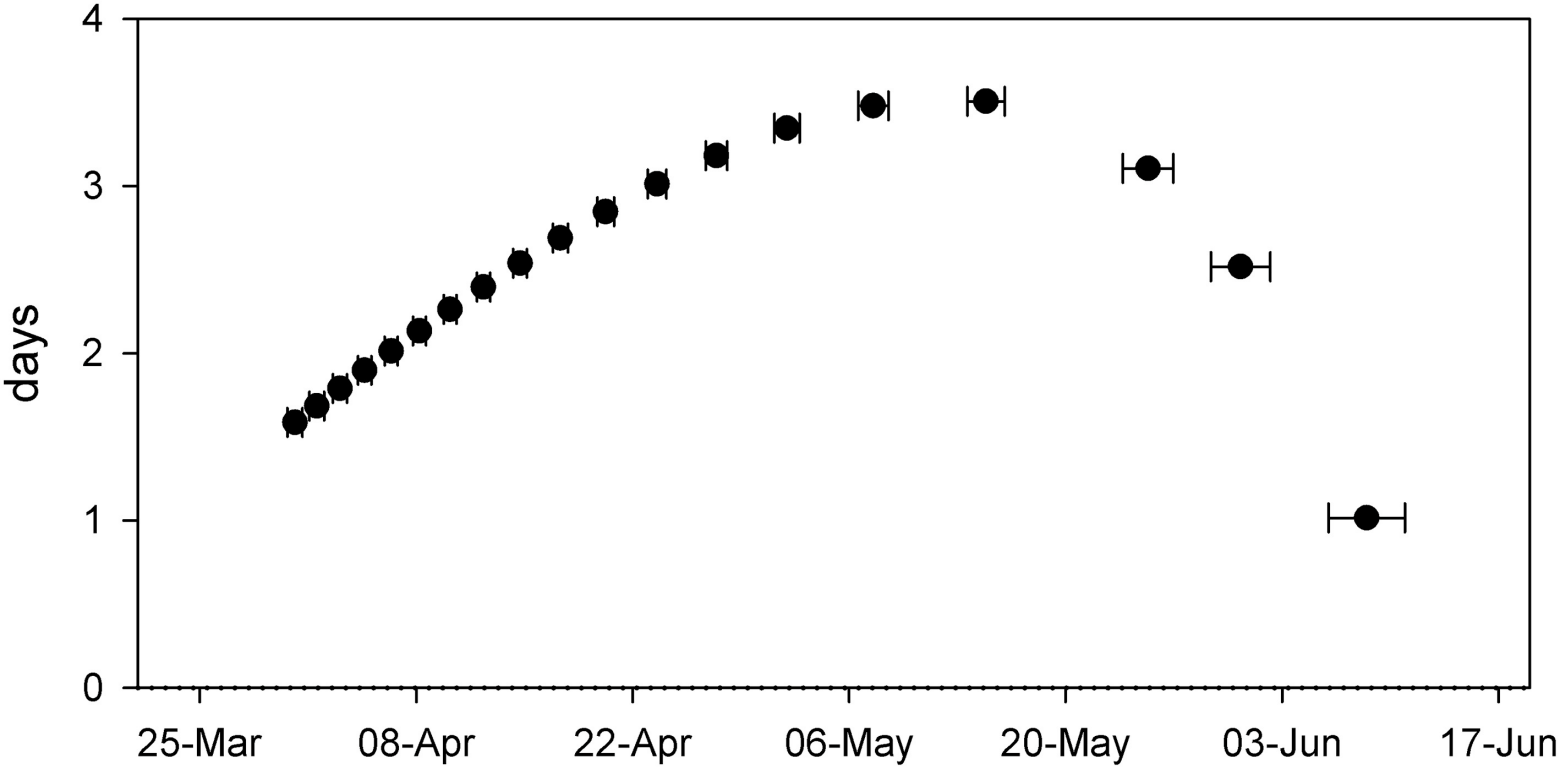
Difference (in days) between the NDVI defined phenological phase of *Phragmites australis* in the western and eastern parts of Lake Balaton at a given date. Each symbol represents an average (±SE, *n* = 9–10) of modeled data derived from the fitted equations.

Comparing resulting phenology from the western and eastern parts of Lake Balaton revealed only minor differences (Figure 4 and Table 1). Although both the NDVI_max_, and the initial rate of NDVI growth (α) were very similar, the results indicated that the plants of the western part of Lake Balaton tended to reach the given phenological stages earlier than the plants from the eastern part of the lake (Figure 4 and Table 1). The peak NDVI increase (GR_max_) in the western (eutrophic) part of Lake Balaton was on the 119th day of the year (29 April), and on the 123th day of the year (3 May) in the eastern (mesotrophic) part (Table 1). The data indicated that the phenological difference between the western and eastern parts of the lake was not consistent, and was observed mainly at the beginning of the vegetation season. The greatest difference, where the western part was 3.5 days ahead of the eastern part of the lake, was observed on 16 May (day 135), and no difference was observed by the middle of July (Figure 5).

**Table 1:** Comparison of maximal seasonal NDVI (NDVI_max_), date of maximal NDVI increase (GR_max_), and the initial rate of NDVI growth (α) calculated from seasonal NDVI measurements of stable and unmowed *Phragmites australis* patches from the western and eastern parts of Lake Balaton.

	**NDVI_max_**	**GR_max_**	**α**
Western	0.691 ± 0.008	118.8 ± 1.0	1.72 ± 0.09
Eastern	0.703 ±0.006	122.9 ± 1.0	1.55 ± 0.06
*t*-test (*t*^P^)	−1.290^ns^	−2.841^*^	1.271^ns^

*Data are average ± SD (n = 9–10)*.

ns *P ≥ 0.05*,

* *P < 0.05*.

The obtained NDVI parameters were also used as a covariate to estimate the effect of the physico-chemical properties of the sediment on phenology of the studied areas (Table 2). Data indicated that the phenological parameters estimated on the basis of NDVI indices correlated not only with trophic (total and nitrate-nitrite nitrogen), but also with the physical properties of the sediment (organic C and clay content, Table 2).

**Table 2:** Pearson product moment correlation (*R*^P^) between the physicochemical properties of the sediment and various phenological parameters of stable and unmowed *Phragmites australis* patches of the northern shore from the western and eastern parts of Lake Balaton.

	**NDVI_max_**	**GR_max_ (day)**	**α**
Organic C (%)	−0.443^ns^	0.748^*^	−0.400^ns^
Total N (g/kg)	0.419^ns^	−0.748^*^	0.383^ns^
(NO_3_+NO_2_)-N (mg/kg)	0.019^ns^	−0.846^*^	0.743^*^
NH_4_-N (mg/kg)	0.176^ns^	0.549^ns^	−0.385^ns^
Soluble P (mg/kg)	−0.138^ns^	−0.133^ns^	−0.046^ns^
Total P (mg/kg)	0.544^ns^	0.412^ns^	−0.135^ns^
Clay content (%)	0.469^ns^	−0.471^ns^	0.783^*^

ns *P ≥ 0.05*,

* *P < 0.05 (n = 7)*.

Due to the bathymetric difference of the studied areas (the western parts are shallower, than the eastern parts of Lake Balaton), the thermal effect on plant phenology was also studied. Comparison of seasonal NDVI changes expressed on the basis of growing day-degrees (not days) indicated no west-east difference in the effect of air or water temperature on NDVI dynamics of the studied reed stands (see Supplementary Figures 2, 3, Supplementary Table 2).

### Stable–die-back comparison

**Figure 6:**
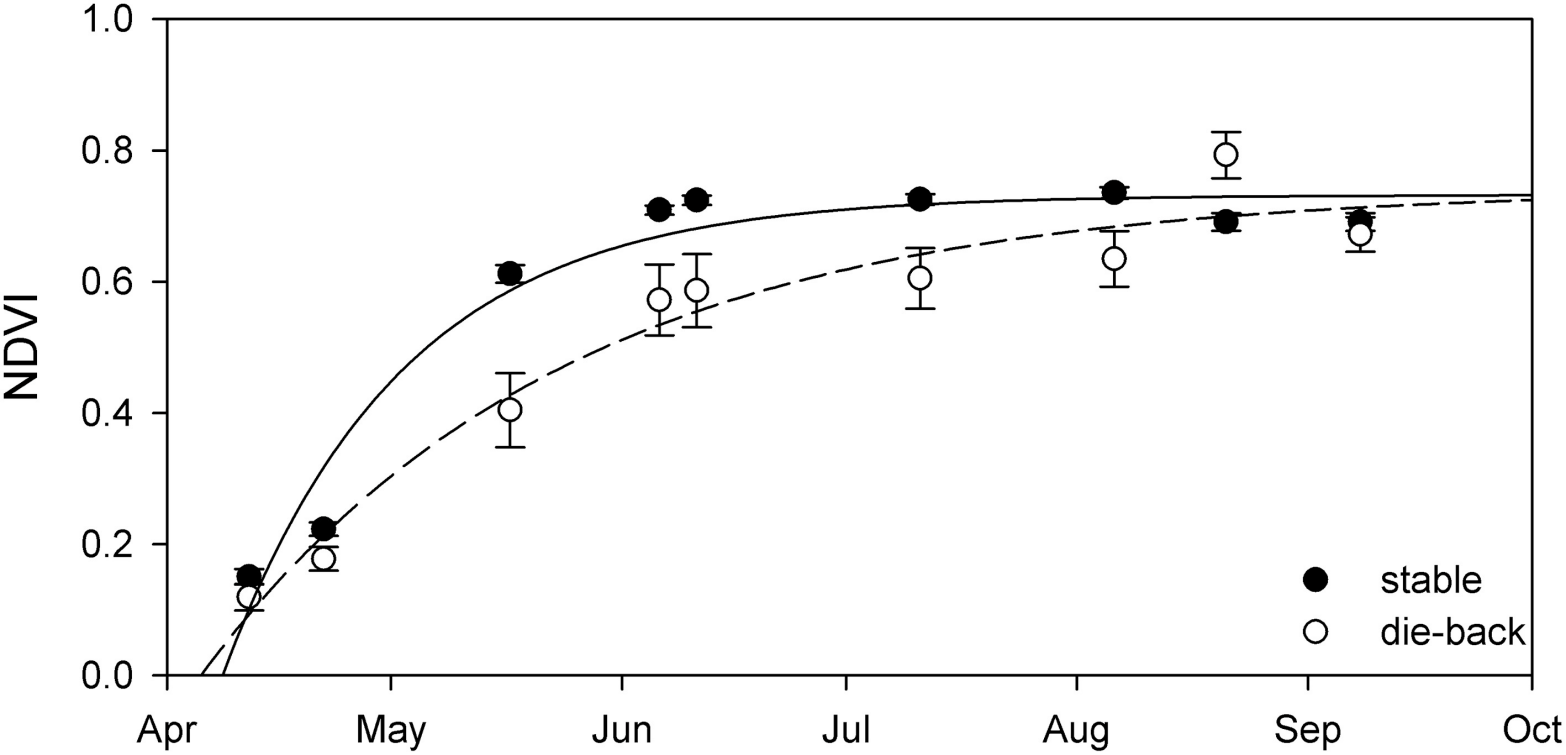
Seasonal change of NDVI in the stable (black circles) and die-back (open circles) stands of Lake Balaton. Data showed are average ± SE (*n* = 3,400 pixels for stable and *n* = 4,650 for die-back). Lines are exponential growth till maximum equation fitted to data.

Comparison of the phenology of stable and die-back *Phragmites* stands of Lake Balaton showed more substantial differences, without any territorial specificity (Figure 6, Table 3). When compared to *Phragmites* from die-back stands, stable reed showed more advanced development, with the plants growing earlier (by ~16 days) and faster (34% higher growth rate - α) in spring (Table 3). Although the estimated maximal seasonal NDVI (NDVI_max_) was higher in the die-back sites (Table 3), higher values were only obtained once for the die-back sites (Figure 6).

**Table 3:** Maximal seasonal NDVI (NDVI_max_), date of maximal intensity growth (GR_max_) and the initial rate of NDVI growth (α) calculated from seasonal NDVI data of stable and die-back *Phragmites australis* plants of Lake Balaton from.

	**NDVI_max_**	**GR_max_**	**α**
Stable	0.698 ± 0.005	121.0 ± 0.8	1.65 ± 0.05
Die-back	0.727 ± 0.013	136.6 ± 5.3	1.09 ± 0.14
*t*-test (*t*^P^)	−2.162^*^	−3.191^**^	3.954^***^

*Data showed are average ±SD (n = 4). ^ns^P ≥ 0.05*,

* *P < 0.05*,

** *P < 0.01*,

*** *P < 0.001*.

To assess physiological underpinnings of the NDVI data, morphological dynamics were compared with NDVI parameters (Table 4, also see Supplementary Figure 4): NDVI GR_max_ was compared with the timing of the maximal intensity of stem, basal diameter, and leaf number growths, while NDVI_max_ was compared to the maximal seasonal value of stem heights, basal diameter, and number of green leaves. A strong relationship between NDVI parameters and the biometric parameter, leaf growth, was discovered, while important morphological parameters related to stem and basal diameter growth have very little, or no correlation to the NDVI (Table 4).

**Table 4:** Pearson product moment correlation (*R*^P^) between various NDVI parameters and dynamics of morphological changes of *Phragmites australis*.

	**NDVI_max_**	**GR_max_**	**α**
Stem growth	0.467^ns^	0.601^*^	–
Basal diameter growth	0.537^ns^	0.290^ns^	0.081^ns^
Leaf number increase	0.918^**^	0.743^*^	0.712^*^

ns *P ≥ 0.05*,

* *P < 0.05*,

** *P < 0.01 (n = 4)*.

**Figure 7:**
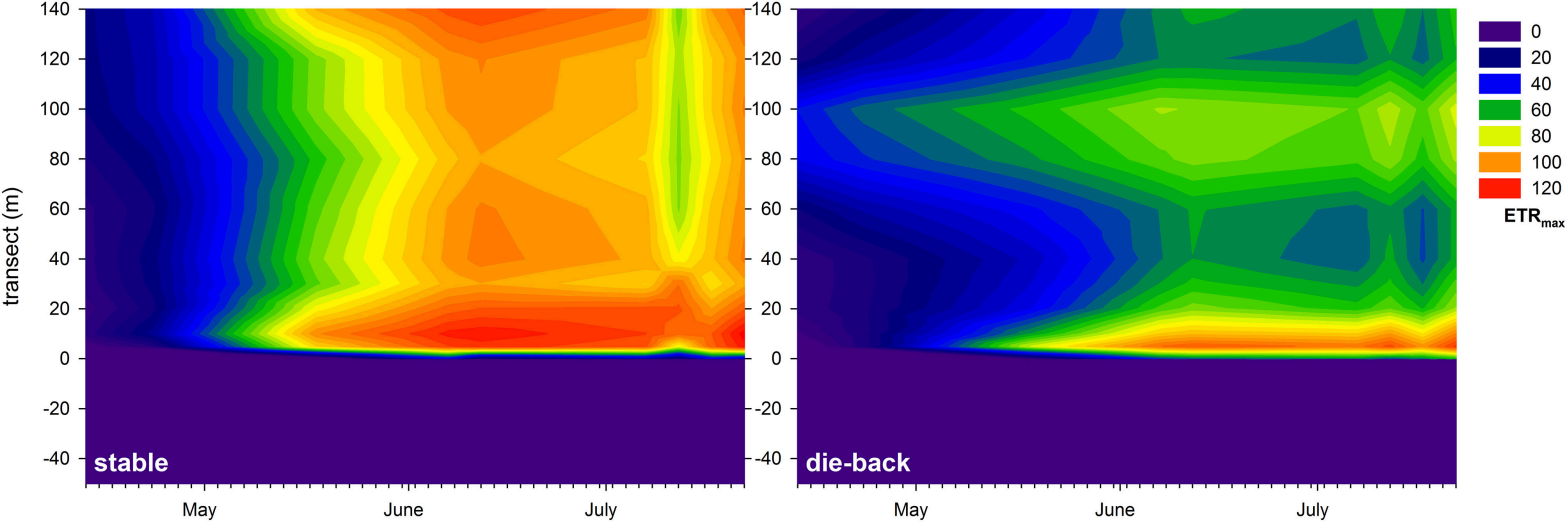
Seasonal changes in the maximal electron transport rate (ETR_max_, relative units) of a 140 m transect in the stable and die-back stands of *Phragmites* in the eastern part of Lake Balaton. The 0 of y axis marks the lakeward side of the stands.

**Figure 8:**
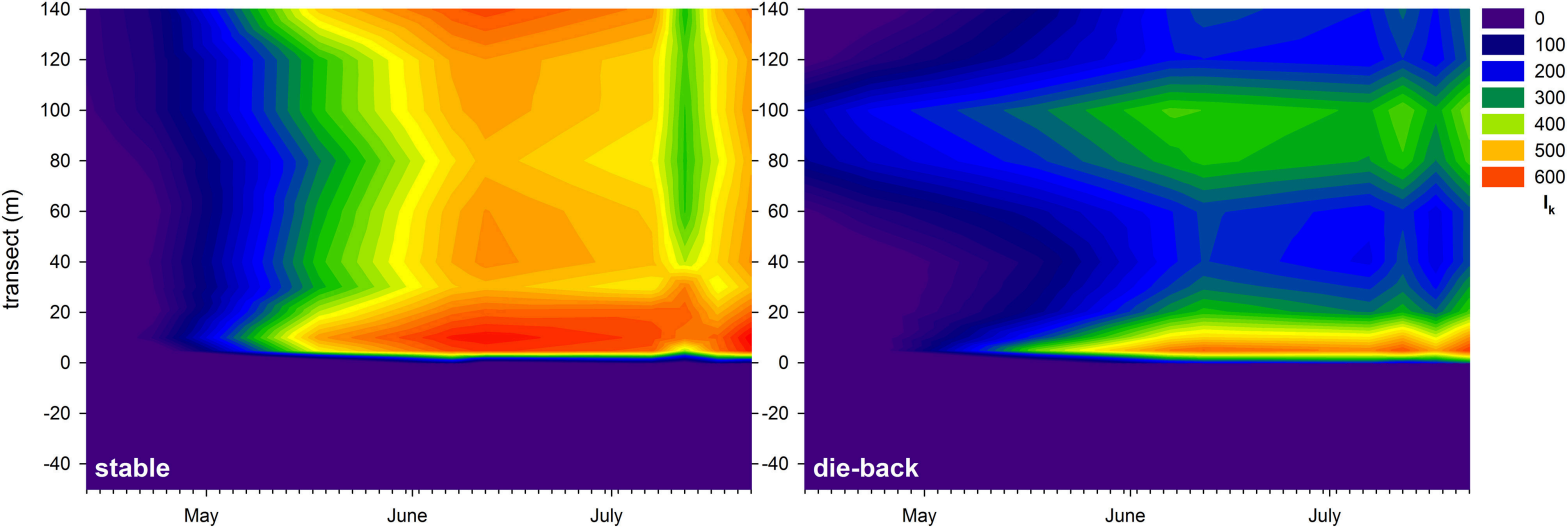
Seasonal changes in theoretical saturation light intensity (I_k_, μmol m^−2^ s^−1^) of a 140 m transect in the stable and die-back stands of *Phragmites* in the eastern basin of Lake Balaton. The 0 of y axis marks the lakeward side of the stands.

**Figure 9:**
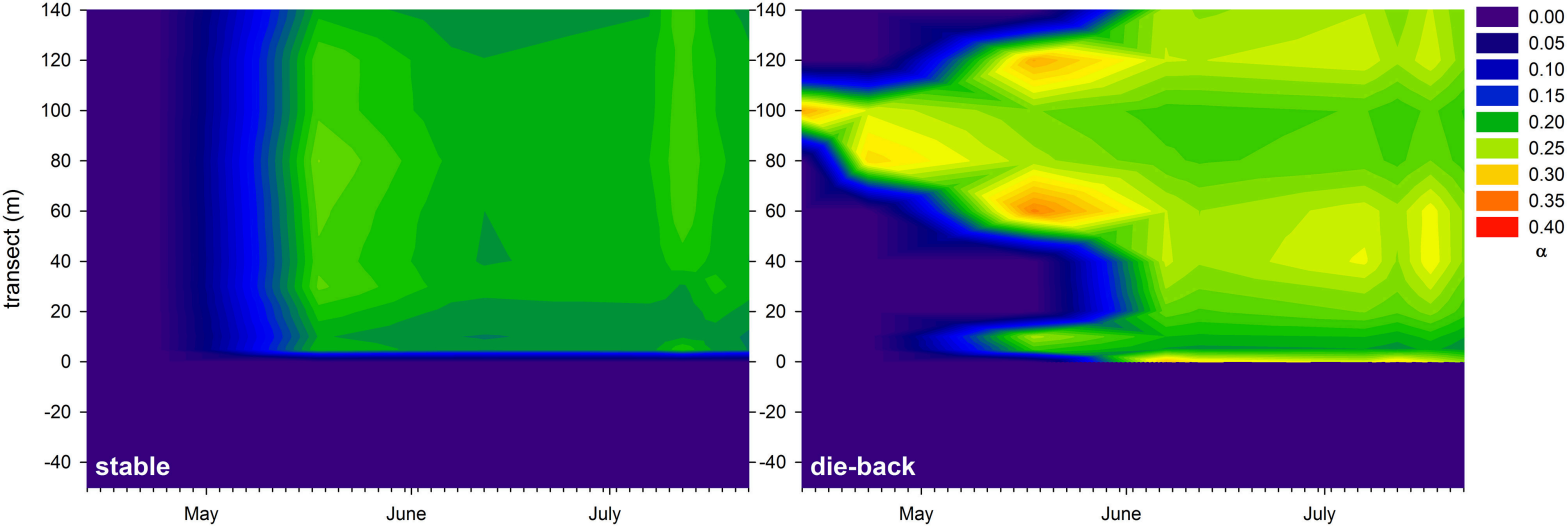
Seasonal changes in the maximum quantum yield for whole chain electron transport (α) of a 140 m transect in the stable and die-back stands of *Phragmites* in the eastern basin of Lake Balaton. The 0 of y axis marks the lakeward side of the stands.

At the stable and die-back sites, both NDVI and photophysiological data were collected. Therefore, following an inter-calibration (Pearson Product Moment Correlation; ETR_max_: *R* = 0.860 *P* < 0.001; I_k_: *R* = 0.841 *P* < 0.001, α: *R* = −0.721, *P* = 0.0011; *n* = 17, also see Supplementary Figure 5), the electron transport rate parameters were visualized for the entire studied transects (Figures 7–9). The differences between the stable and the die-back plants were consistent throughout the studied reed stands (i.e., the stable stands had higher ETR_max_ — more vigorous photosynthetic capacity (Figure 7)—as well as with higher I_k_—higher need for light (Figure 8)—and lower α–slower response to irradiance (Figure 9)—then the die-back stands in most cases. Despite these differences, the studied stands also have commonalities: throughout the vegetation season, high photophysiological parameters were recorded for plants at the lakeward (2–28 m of the transects) and terrestrial sides (stable ~140 m, die-back 80–100 m of the transects) of both the stable and die-back stands (Figures 7–9).

### Mowed–not mowed comparison

**Figure 10:**
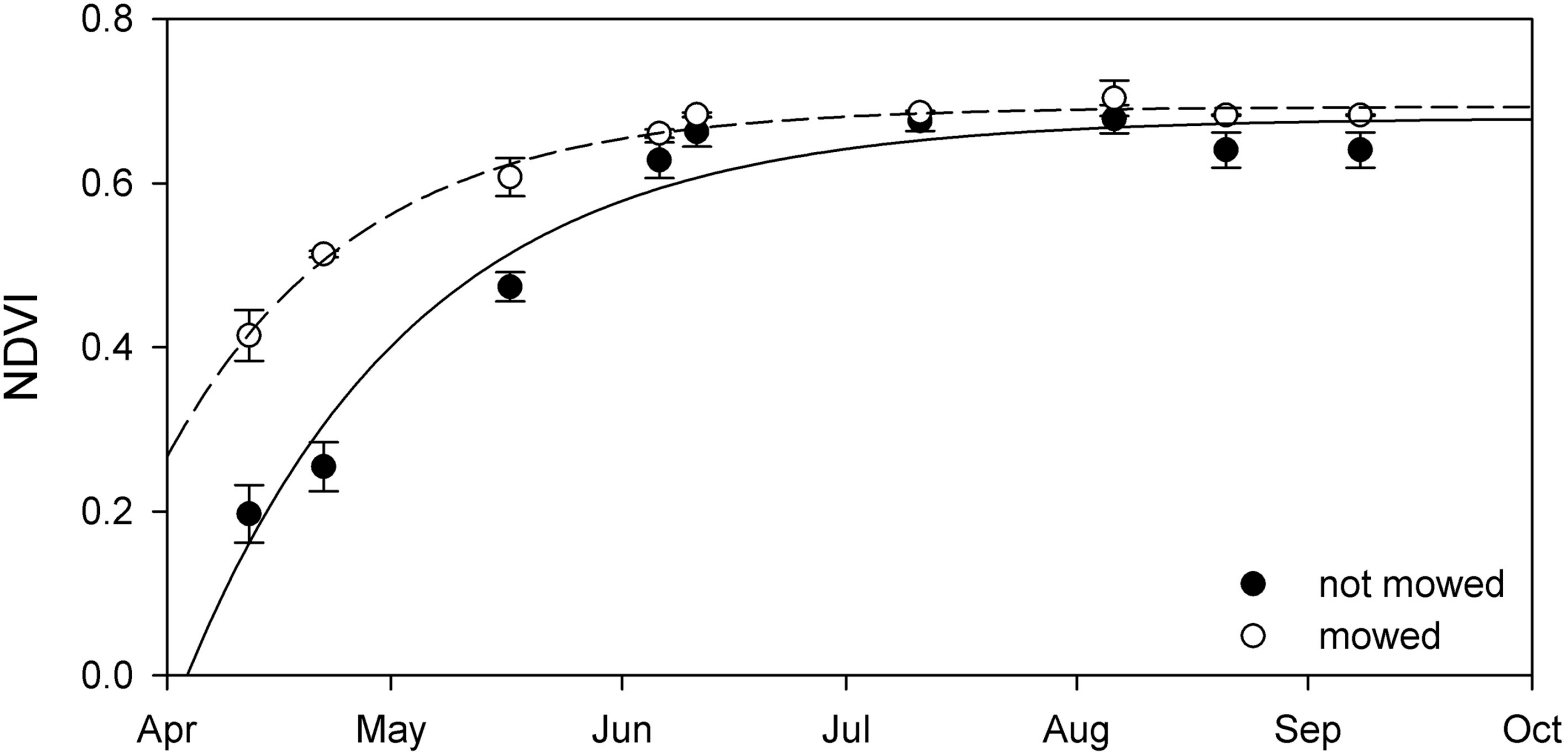
The seasonal change of NDVI from not mowed (black circle) and mowed (open circle) patches of stable *Phragmites australis* in Lake Balaton. Each symbol represents the mean (±SE, *n* = 2,100 pixels for mowed and *n* = 2,700 pixels for not mowed). Lines are exponential growth till maximum equations fitted to the data.

The mowed stands had significantly higher NDVI until July (Figure 10). Further analysis indicated that plants at the mowed patches reached maximal NDVI growth 16 day earlier, in middle of April (Table 5). It impossible to assess whether this difference is based on advanced biomass accumulation or the lack of dry reed in the mowed patches since biomass was not sampled at these sites.

**Table 5:** Maximal seasonal NDVI (NDVI_max_), date of maximal intensity growth (GR_max_) and the initial rate of NDVI growth (α) calculated from seasonal NDVI data of mowed and not mowed reed stands of Lake Balaton from.

	**NDVI_max_**	**GR_max_**	**α**
Mowed	0.692 ± 0.005	103.9 ± 1.1	1.70 ± 0.07
Not mowed	0.675 ± 0.010	119.9 ± 0.5	1.58 ± 0.09
*t*-test (*t*^P^)	0.0174^ns^	−16.022^***^	0.813^ns^

*Data showed are average ± SD (n = 2)*.

ns *P ≥ 0.05*,

*** *P < 0.001*.

## Discussion

The first objective of this study was to evaluate the potential of using UAVs to assess aquatic vegetation. UAV platforms have several desirable properties. Flexibility of use (transportable and operable by a single person) allows the researcher to use it as soon as the weather and other conditions allow. By adjusting the flight height, the spatial resolution of the survey can be optimized and regulated, although there is a tradeoff between increased spatial resolution (i.e., pixel size) and area covered by a given overpass. It quickly became obvious that sub-decimetre resolution images obtained with Tetracam ADC Lite allowed identification of only major groups of vegetation, like shrubs, *Phragmites*, and herbaceous riparian vegetation. This was the result of a pronounced rolling shutter effect of the camera that consequently ensued necessity of obtaining more than one image from the same site. Nevertheless, a better, high resolution camera could accurately identify vegetation type even at the species level. This study demonstrates that use of UAV as a remote sensing tool enables limnologists and plant scientists to quantify ecological processes at significantly larger areas, both at individual plant and at community levels.

The second objective of this study was to assess the potential of performing repeat measurements at the same spot throughout the vegetation period (i.e., season), so as to estimate the seasonal dynamics of plant growth (phenology). The semi-automated operation of the given UAV platform allowed the repeated visit of the assessed area, thus considerably simplifying these measurements. Unfortunately, the periodically low accuracy of the GPS signal originated from satellite geometry, user range error, and local factors such as signal blockage, atmospheric conditions, and receiver design features/quality resulted of the assessment of a different reed stand, sometimes up to 40–60 m away from the original plot. However, this limitation was overcome by surveying a larger area so as to increase the chances of overlapping sampling areas. This manuscript used NDVI-based phenology metrics (parameters describing the dynamics of NDVI changes) that are distinct from the familiar metrics used in classical phenology (onset of growth, timing of blooming, start and end of senescence). However, even the use of descriptors based on biomass accumulation showed that at small (within-stand) and medium (between-stand) scales the use of UAV-based NDVI vegetation assessment is practicable.

Our understanding of phenology is good, although there are differences between the observations, therefore understanding the source of these differences between the phenological observations are important. Comparison of *Phragmites* phenology in Lake Balaton showed that sites in the western (eutrophic) part reached certain phenological stages 3.5 days earlier than sites in the eastern (mesotrophic) part of the lake. This difference in phenology is relatively large and in the region similar data were reached during the last 150 year as a result of climate change (Walkovszky, 1998; Molnár et al., 2012; Szabó et al., 2016). The scientific community is in agreement that temperature is an important driver of phenological shifts, especially in case of climate change (Estrella et al., 2009; Szabó et al., 2016), although other factors, such as CO_2_ and trophic change may also play a significant role (Clevering et al., 2001; Cleland et al., 2006; Tylová et al., 2008). The data presented here also suggest that the observed phenological difference between the reed of the western and eastern parts of Lake Balaton is related to nitrogen content of the sediment (total nitrogen and nitrate-nitrite content). Although this is contrary to previously published results on other species (Cleland et al., 2006; Nord and Lynch, 2009; Xia and Wan, 2013), results of the study show *Phragmites* in nitrogen rich sediments reaches some phenological stages earlier. This result has been observed elsewhere (Cleland et al., 2006), however, the extent of the observed phenological shift implies a heightened sensitivity of macrophytes to the trophic status of the sediment. Beside this trophic effect, *Phragmites* phenology also correlated with the physical state (consistency) of the sediment (clay and organic C content). While phenological differences between the eastern and western basins of Lake Balaton were apparent, the difference between the phenology of stable and die-back *Phragmites* was even more substantial; die-back plants reached phenological milestones on average 15.6 days later than stable plants. This fits well with previously published results of *Phragmites* die-back in Lake Balaton and in numerous other European lakes (Den Hartog et al., 1989; Ostendorp, 1989; Tóth, 2016). In summary, UAVs are potentially very useful tools for the assessment of vegetation phenology, or any kind of repeated measurements throughout the vegetation period, even in difficult-to-access areas, such as wetlands or high canopy trees.

Besides the difference between the phenology of stable and die-back stands (11 days in the western and 21 days in the eastern part of the lake), differences within the stands were also observed. Both the NDVI data and the correlated photophysiological parameters revealed the hidden patterns of primary production within the reed stands. The data indicated not only an overall decrease in electron transport in die-back plants, suggesting decreased primary production of these *Phragmites* plants as previously described elsewhere (Fogli et al., 2002; Mészáros et al., 2003), but also showed a spatial pattern (western vs. eastern sites), and a temporal (seasonal) dynamic as well. Moreover, the electron transport of *Phragmites* at the die-back sites saturated faster and at lower light intensities. Primary production of *Phragmites* in both stable and die-back stands had two hotspots (i.e., two photophysiologically active regions); at the lakeward and at the terrestrial sides. These areas are favorable coincidences of internal and external factors at local, 15–25 m wide areas that stimulate the production of reed and might be the positions of the deepwater or terrestrial reed ecotypes (Tóth and Szabó, 2012). The UAV platform therefore revealed zonation within a *Phragmites* stand (i.e., a clonal adaptation to a particular set of environmental conditions in areas where recolonization via seed dispersal is not possible; Koppitz, 1999; Engloner et al., 2010; Engloner and Major, 2011; Tóth and Szabó, 2012). Clonal specificities of *Phragmites*, for example intraclonal cooperation (physiological integration of ramets), and interclonal competition (Pitelka and Ashmun, 1985; Hara et al., 1993; Kroon, 1993) could explain the observed size and position of the photophysiologically active areas. The zonation observed using this technique may help to understand how interclonal competition and intraclonal cooperation might affect boundaries and zonation of *Phragmites*. Moreover, it might be useful in studies about the macrophyte environmental adaptations in multispecies wetlands.

This study demonstrated the possibility of using UAVs as a flexible remote sensing platform. By means of an NDVI camera, not only a quick and useful vegetation biomass estimation tool is available, but with multiple repeat measurements throughout the season, phenology of the study sites could be estimated and compared, while large area coverage allowed nuanced features in sub-site zonation to be revealed. Both techniques (i.e., the phenological and the microvariation assessment) could help plant scientists to understand how vegetation changes in time and space with respect to temporal and site-specific conditions. On the other hand, it could supply invaluable information to conservation specialists regarding the abundance, condition, and threats to conservation in natural ecosystems.

## Author contributions

The author confirms being the sole contributor of this work and approved it for publication.

### Conflict of interest statement

The author declares that the research was conducted in the absence of any commercial or financial relationships that could be construed as a potential conflict of interest.
