# Supplementary material for: Monitoring Spatial Variability and Temporal Dynamics of Phragmites Using Unmanned Aerial Vehicles
Source: Front Plant Sci. 2018 Jun 4;9:728. doi: 10.3389/fpls.2018.00728 (PMC5994432; doi:10.3389/fpls.2018.00728)
Supplement: Supplementary file 1 [file Image_1.PDF]

Supplementary Material

Monitoring spatial variability and temporal dynamics of Phragmites using unmanned aerial vehicles

Viktor R. Tóth

Correspondence: Viktor R. Tóth: toth.viktor@okologia.mta.hu

1.1 Supplementary Figures

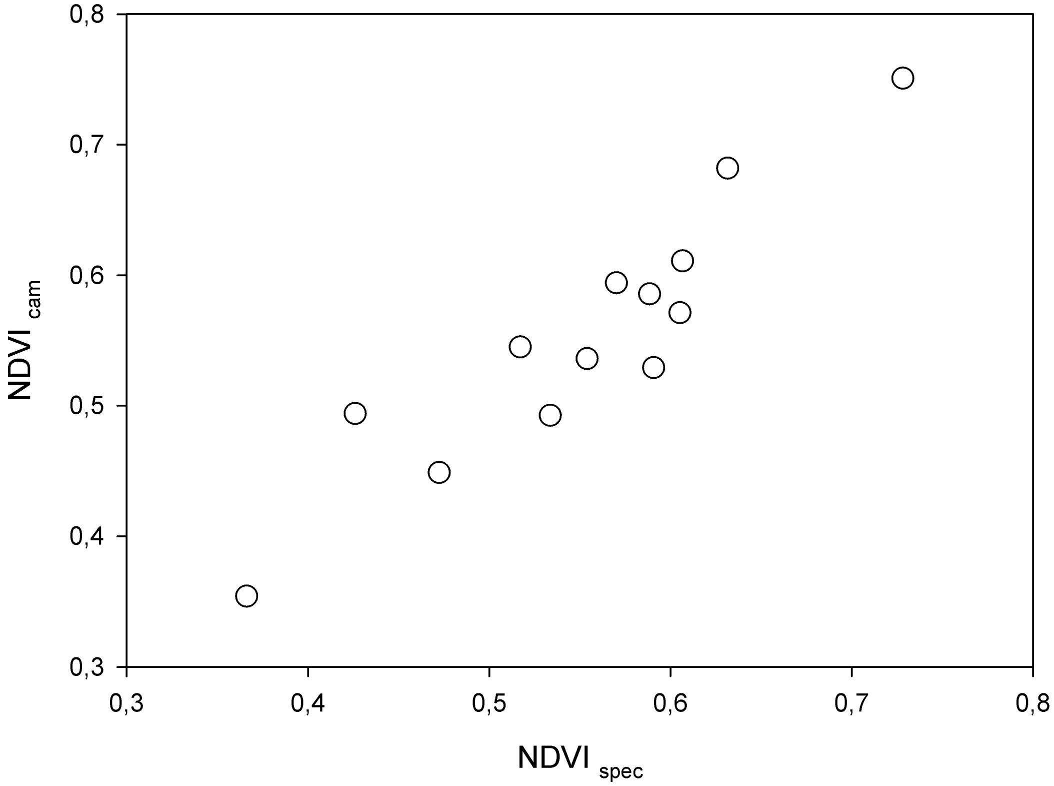

Supplementary Figure 1. Validation of the NDVI data obtained from camera ($NDVI_{cam}$) with the NDVI data calculated from spectroradiometric data ($NDVI_{spec}$). Pearson product moment correlation $r=0.928$, $P<0.001$.

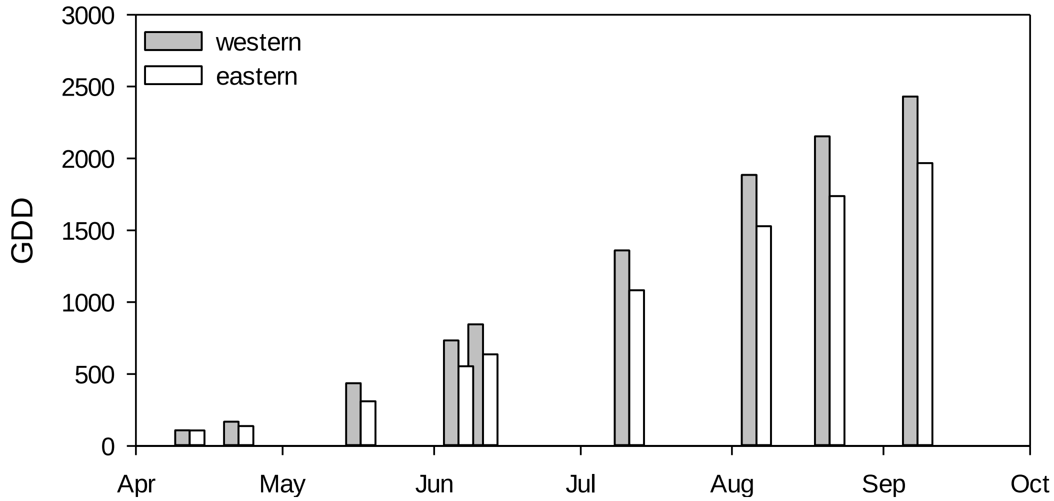

Supplementary Figure 2. Growing degree day in the western and eastern parts of Lake Balaton in 2014. Growing degree days were calculated as $\boldsymbol{GDD = \sum([(T_{max} + T_{min})/2] - T_0)}$, where T_0 is the base temperature regarded as a temperature of reed emergence (7°C), while T_{max} and T_{min} are the daily maximal and minimal temperatures.

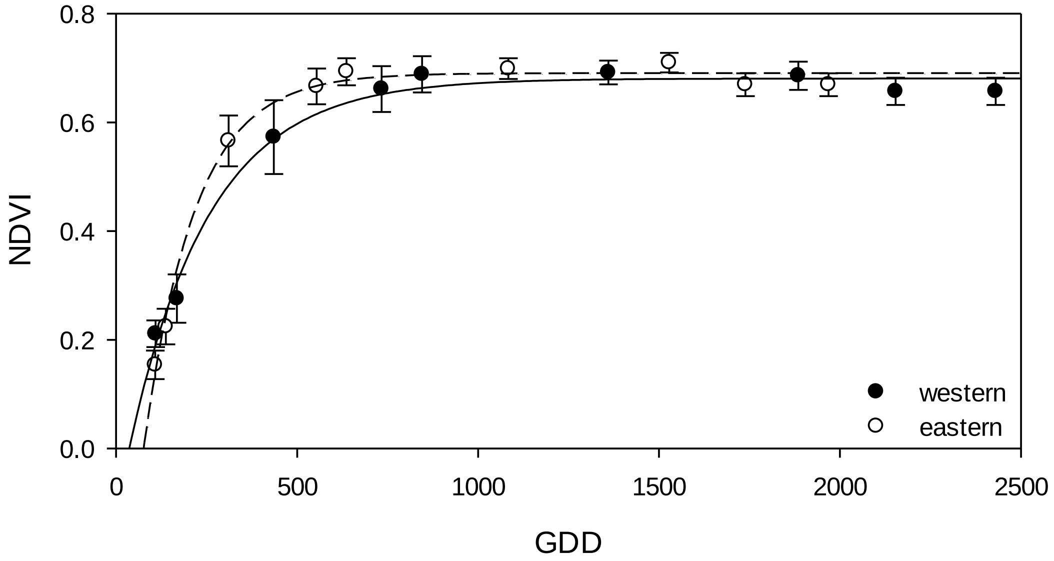

Supplementary Figure 3. Change of the NDVI of stable, unmowed *Phragmites australis* as a function of growing degree days (GDD) from the western and eastern parts of Lake Balaton. Each symbol represents an average (±SE, n=9100-12100) of the exponential growth till maximum fitted to each separate site. For statistics see **Supplementary Table 2**.

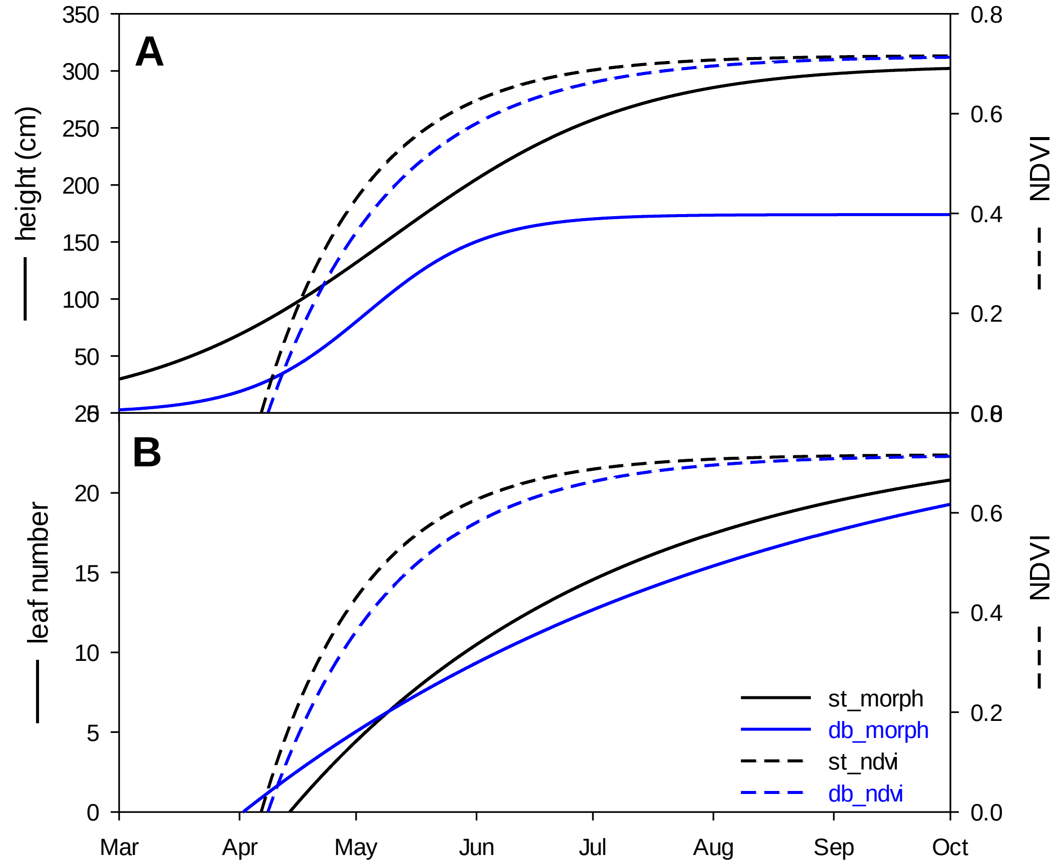

Supplementary Figure 4. Relation between *Phragmites australis* morphological and NDVI data. **A.** Seasonal change of stem height (solid line) and NDVI data (dashed line) at the stable (st) and die-back (db) reed stands (Pearson product moment correlation between the actual heights and NDVI data was r=0.467, P=0.290 and r=0.537 P=0.217 for stable and die-back stands, respectively). **B.** Seasonal change of leaf number (solid line) and NDVI data (dashed line) at the stable (st) and die-back (db) reed stands (Pearson product moment correlation between the actual leaf number and NDVI data was (r=0.743, P=0.0458 and r=0.918 P=0.00353 for stable and die-back stands, respectively).

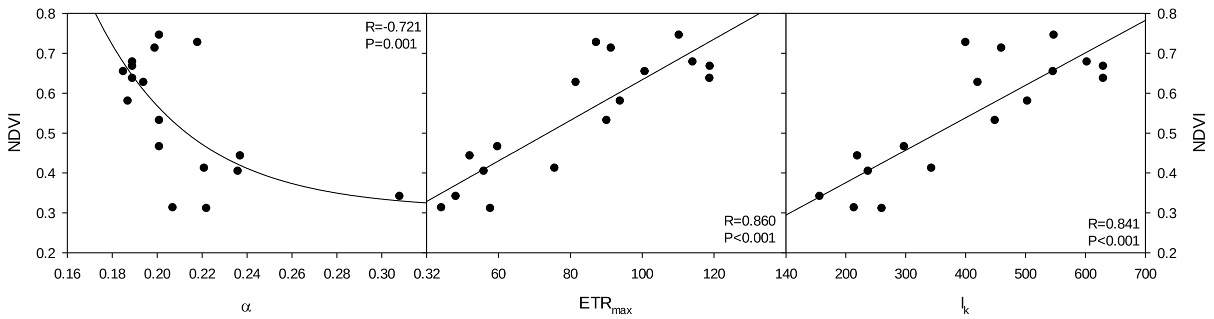

Supplementary Figure 5. Correlation between key photochemical parameters of the PSII electrontransport chain of *Phragmites australis* measured by pulse amplitude modulated fluorescence and their respective NDVI data. Results of the Pearson product moment correlations are shown on the graphs.

1.2 Supplementary Tables

Supplementary Table 1. Location (northing coordinate - N, easting coordinate - E), situation within Lake Balaton (area), ecological status (stable vs. die-back), mowed or not, ground pixel size (gps - cm), flight height (m), transect measurement (tran), chlorophyll fluorescence measurement (PAM), sampling for sediment physicochemical properties (sediment) and biometric analysis of plants (morpho) of the selected *Phragmites* stands.

	N	E	area	status	mowed	gps	height	tran	PAM	sediment	morpho
**1**	46° 46' 54.01"	17° 22' 55.34"	west	die-back		4.0-4.6	100-115		yes	yes	
**2**	46° 46' 57.90"	17° 23' 02.29"	west	die-back		4.0-5.0	100-125	yes	yes	yes	yes
**3**	46° 47' 45.02"	17° 24' 48.45"	west	stable		4.2-4.8	105-120	yes	yes	yes	yes
**4**	46° 47' 39.64"	17° 25' 09.78"	west	stable		4.2-5.1	105-128				
**5**	46° 47' 37.96"	17° 25' 14.30"	west	stable		4.2-4.8	105-120				
**6**	46° 47' 36.02"	17° 25' 19.88"	west	stable		4.4-5.0	110-125				
**7**	46° 47' 32.89"	17° 25' 26.68"	west	stable	yes	4.0-4.6	100-115				
**8**	46° 42' 50.72"	17° 20' 59.46"	west	stable		4.4-5.0	110-125				
**9**	46° 42' 45.53"	17° 21' 18.64"	west	stable		4.4-5.0	110-125				
**10**	46° 42' 22.71"	17° 24' 36.03"	west	stable		4.2-4.9	105-123				
**11**	46° 42' 27.61"	17° 25' 48.03"	west	stable		4.2-5.2	105-130				
**12**	46° 42' 29.08"	17° 26' 06.57"	west	stable		4.0-4.6	100-115				
**13**	46° 54' 47.87"	17° 49' 57.10"	east	stable		4.2-4.9	105-123		yes	yes	
**14**	46° 55' 02.89"	17° 50' 20.75"	east	die-back		4.0-4.6	100-115		yes	yes	
**15**	46° 51' 40.39"	17° 53' 30.22"	east	stable		4.2-4.9	105-123				
**16**	46° 51' 42.08"	17° 53' 38.72"	east	stable		4.2-5.2	105-130				
**17**	46° 57' 52.27"	17° 55' 02.67"	east	die-back		4.2-5.2	105-130	yes	yes	yes	yes
**18**	46° 58' 06.20"	17° 55' 13.04"	east	stable		4.2-4.9	105-123	yes	yes	yes	yes
**19**	46° 58' 05.66"	17° 55' 15.49"	east	stable		4.2-4.9	105-123				
**20**	46° 58' 04.97"	17° 55' 20.27"	east	stable		4.2-4.6	105-115				
**21**	46° 58' 29.74"	17° 56' 38.00"	east	stable		4.4-5.0	110-125				
**22**	46° 58' 32.84"	17° 56' 38.68"	east	stable		4.4-5.2	110-130				
**23**	46° 58' 27.29"	17° 57' 47.05"	east	stable	yes	4.4-5.2	110-130				
**24**	46° 58' 27.37"	17° 57' 51.26"	east	stable		4.8-5.2	120-130				
**25**	46° 59' 35.37"	17° 59' 30.65"	east	stable		4.4-5.6	110-140				

Supplementary Table 2. Maximal seasonal NDVI ($NDVI_{max}$), date of maximal intensity growth (GR_{max}) and the initial rate of NDVI growth (α) calculated from seasonal NDVI data as a function of growing degree-days (GDD) of stable, unmowed *Phragmites australis* plants from the western and eastern parts of Lake Balaton. Data showed are average±SE (n=9-10). P: ns - P≥0.05, * - P<0.05.

	$NDVI_{max}$	GR_{max}	α
**western**	0.684±0.007	120.3±0.4	0.178±0.018
**eastern**	0.688±0.006	120.3±0.6	0.279±0.028
**t-test (t^P)**	-0.004^{ns}	-0.475^{ns}	-2.583^{*}
